# Imaging and pathological diagnosis of primary intracranial malignant melanoma: A case report and literature review

**DOI:** 10.1097/MD.0000000000032767

**Published:** 2023-02-03

**Authors:** Wei-Li Long, Fu-Yong Chen, Xiao-Lin Huang, Jun-Xu Lu, Yan-Neng Xu

**Affiliations:** a Department of Pathology, Luzhou People’s Hospital, Luzhou, Sichuan, China; b Department of Radiology, Luzhou People’s Hospital, Luzhou, Sichuan, China; c Department of Interventional Radiology, The Affiliated Traditional Chinese Medicine Hospital of Southwest Medical University, Luzhou, China.

**Keywords:** gene mutation, intracranial malignant melanoma, pathology, primary, the differential diagnosis

## Abstract

Primary intracranial malignant melanoma (PIMM) is a rare malignant tumor that lacks specific clinical manifestations. Preoperative diagnosis is difficult to differentiate from meningiomas on computed tomography (CT) scans. Magnetic resonance imaging (MRI) usually shows typical characteristics with high signal intensity on T1WI and low signal intensity on T2WI. PIMM is highly invasive, insensitive to chemoradiotherapy, and has a poor prognosis.

**Patient concerns:**

A 27-year-old woman was admitted to the hospital with a headache for 10 days. She did not experience nausea, vomiting, dizziness, or any other discomfort. A computerized tomography (CT) scan demonstrated a high-density mass in the left cerebellum with patchy calcification at the posterior edge, and heterogeneous enhancement was observed on a contrast-enhanced scan. MRI revealed typical characteristics of high signal intensity on T1WI and low signal intensity on T2WI. The signal characteristics of FLAIR were similar to those of T2WI, and diffusion-weighted imaging (DWI) sequence showed limited diffusion of the tumor. Magnetic resonance spectroscopy revealed increased choline (Cho) and decreased creatine (Cr) and N-acetyl aspartate (Naa) in the tumor.

**Interventions:**

The patient underwent tumor resection and postoperative chemoradiotherapy and immunotherapy.

**Pathological diagnosis:**

Histological and Immunohistochemistry (IHC) tests confirmed the diagnosis of PIMM. In addition, genetic testing revealed GNAQ gene variation.

**Outcomes:**

No recurrence or complications were observed during the follow-up for 6 months.

**Lessons:**

PIMM is rare, and its pathological diagnosis should be closely combined with clinical and medical history. GNAQ is a common variant of PIMM and is expected to be a therapeutic target.

## 1. Introduction

Primary intracranial malignant melanoma (PIMM) is highly malignant and is insensitive to radiotherapy and chemotherapy. In addition, it lacks specificity in clinical manifestations, so it is easily confused with meningioma and other tumors before an operation.^[1]^ Currently, most cases are reported as individual and minor cases. The 2016 World Health Organization Classification of central nervous system tumors classifies them as melanocytosis, melanomas, malignant melanomas, and melanomas,^[2]^ of which malignant melanoma is sporadic. This paper reports the clinical diagnosis, treatment, imaging characteristics, pathological features, and differential diagnosis of a case of PIMM to deepen our understanding of this disease.

## 2. Case description

**Figure 1.**
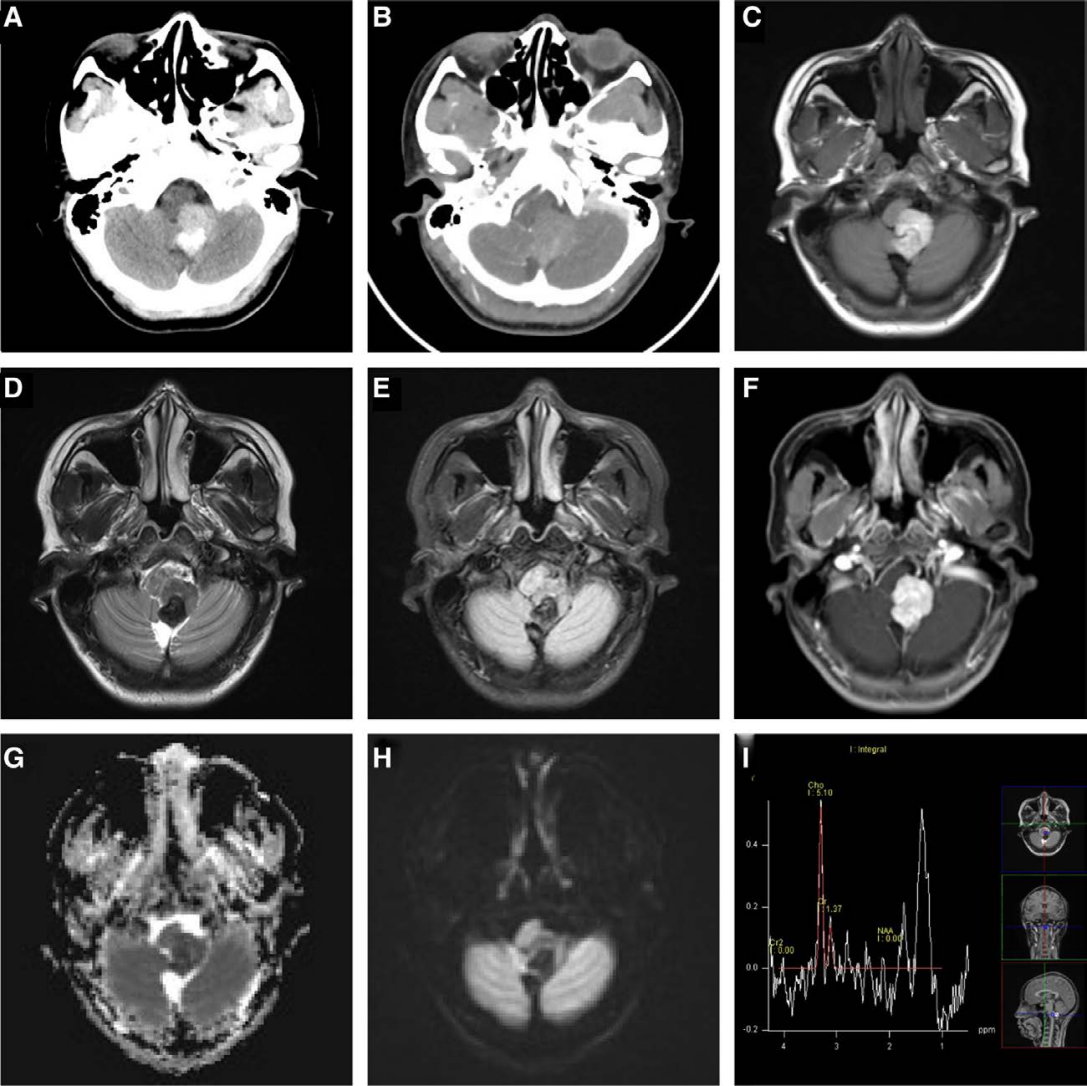
**(a, b**) CT scan demonstrated a high-density mass in the left cerebellum with patchy calcification at the posterior edge, and heterogeneous enhancement underwent a contrast-enhanced scan. **(c, d**) MRI revealed high signal intensity on the T1WI and low signal intensity on the T2WI. **(e**) The signal characteristics of the FLAIR were like T2WI. **(f**) The tumors showed obvious homogeneous enhancement during a contrast-enhanced scan on the T1WI sequence. **(g, h**) The diffusion-weighted imaging (DWI) sequence showed limited diffusion of the tumor. **(i**) MRS Showed increased Choline (Cho) and decreased Creatine (Cr) and N-acetyl aspartate (Naa) in the tumor. CT = computerized tomography, MRI = magnetic resonance imaging, MRS = magnetic resonance spectroscopy.

A 27-year-old woman was admitted to our hospital with a headache for 10 days. Ten days prior, the patient experienced a headache without obvious inducement, which was not relieved after rest. She did not experience nausea, vomiting, dizziness, or any other discomfort. The patient visited the outpatient department of a local hospital. A computerized tomography (CT) scan demonstrated a high-density mass in the left cerebellum with patchy calcification at the posterior edge, and heterogeneous enhancement was observed on a contrast-enhanced scan (Fig. 1a and b). Magnetic resonance imaging (MRI) revealed a high signal intensity on T1WI and low signal intensity on T2WI (Fig. 1c and d). The tumor showed noticeable homogeneous enhancement on contrast-enhanced T1WI (Fig. 1f). The signal characteristics of FLAIR were similar to those of T2WI (Fig. 1e), and the diffusion-weighted imaging (DWI) sequence showed limited diffusion of the tumor (Fig. 1g and h). Magnetic resonance spectroscopy revealed increased choline (Cho) levels. It decreased the creatine (Cr) and N-acetyl aspartate (Naa) levels in the tumor (Fig. 1i). Physical examination revealed that the general condition of the patient was good, and the patient’s mind was clear. Both pupils were approximately 3 mm in size, and light reflection from the eyes was regular. Muscle tension of the extremities was expected, and the pathological signs, finger-to-nose test, and Heel-knee-Shin test results were negative. There was no history of melanoma of the skin, mucosa, or eyes. The results of the laboratory tests could have been unusual.

**Figure 2.**
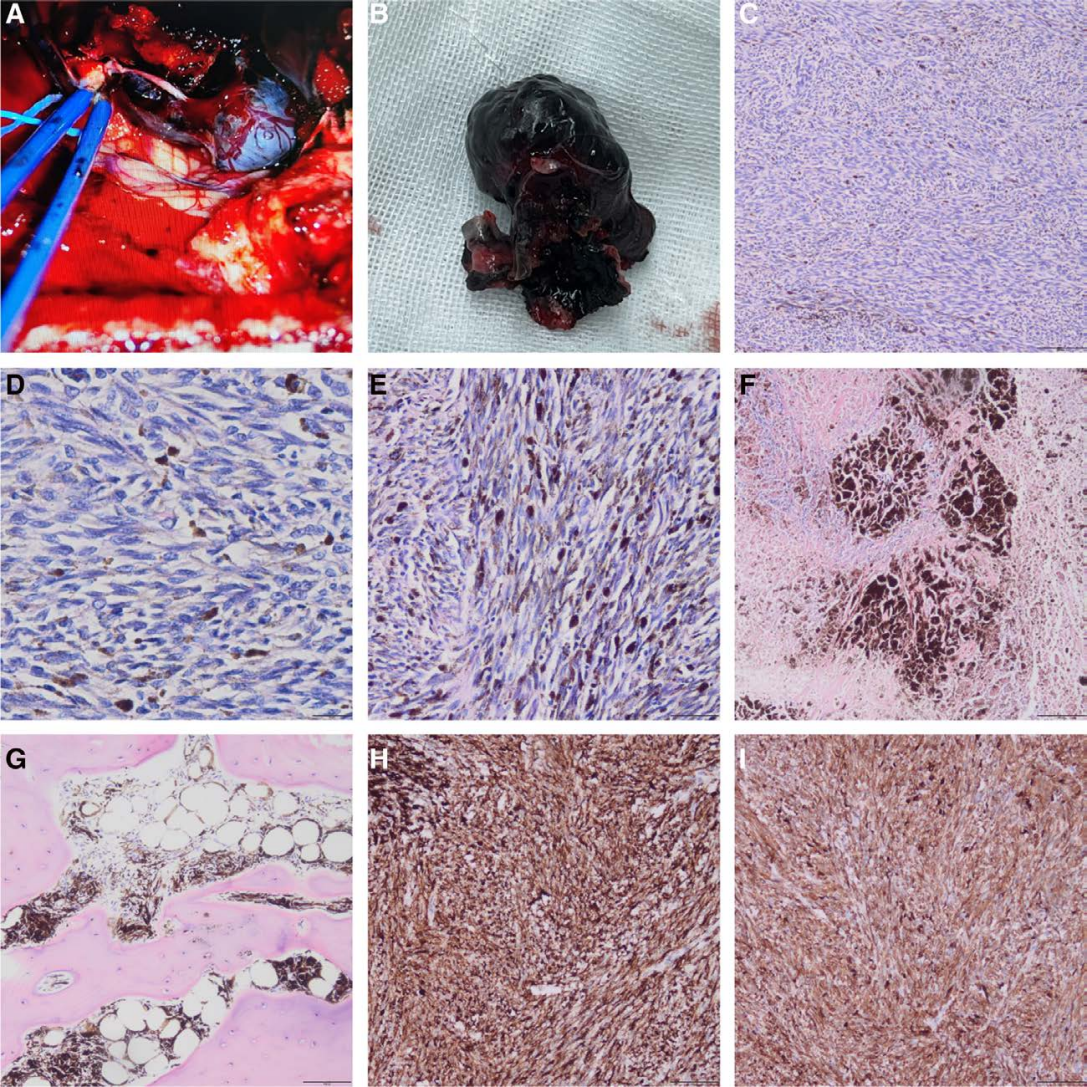
**(a**) During the operation, a black mass on the left side of the vermis of cerebellum was observed. **(b**) Gross image of the tumor. **(c**) The tumor cells were arranged in sheets and bundles (×100, HE). **(d**) Edges of chromatin and prominent eosinophilic nucleoli (×400, HE). **(e**) A wide range of cells contained a large amount of black pigment granules (×200, HE). **(f**) The emorrhage and necrosis were seen (×100, HE). **(g**) The skull showed tumor invasion (×100, HE). **(h**)Tumor cells were diffusely positive for HMB-45 (×100, IHC). **(i**) Tumor cells were diffusely positive for Melan-A (×100, IHC). HE = Hematoxylin-Eosin staining, IHC = immunohistochemistry.

Intraoperatively, the dural tension was high, and a dark hard mass was observed on the left side of the cerebellar vermis (Fig. 2a). The dura mater and foramen magnum near the tumor are dyed black. The tumor, dark meninges, and dark bone tissues were completely resected and sent for pathological examination.

Macroscopic observation showed a solid, black, and slightly hard tumor with a clear boundary, about 3.0 × 2.5 × 2.3 cm^3^ in size (Fig. 2b). The adjacent dura and skull were black stained. Microscopic examination revealed that the tumor area was composed of spindle and oval epithelioid cells arranged in sheets and bundles (Fig. 2c). These cells were moderately heterotypic, with prominent eosinophilic nucleoli. Chromatin was found to be distributed at the cell edge (Fig. 2d). The cytoplasm was abundant and a wide range of cells contained many black pigment granules (Fig. 2e). A large amount of pigmentation was observed between the cells. Hemorrhage and necrosis were observed in some areas (Fig. 2f). The dura and skull were invaded by tumor cells (Fig. 2g). Immunohistochemistry (IHC) showed diffuse positivity for HMB-45 (Fig. 2h), Melan-A (Fig. 2i), S-100, and Vimentin, but negative for pCK, EMA, and GFAP. Ki-67 hotspots were approximately 15%. Genetic tests showed GNAQ gene variation, whereas BRAF, BRCA2, CDK4, CDKN2A, CTNNB1, GNA11, KIT, MAP2K1, NF1, NRAS, PTEN, and TP53 gene variations were not detected. Pathological diagnosis confirmed that the tumor was a primary malignant melanoma of the foramen magnum.

The patient received postoperative chemoradiotherapy and immunotherapy, and no recurrence or complications were observed during the follow-up for 6 months.

## 3. Discussion

PIMM is a rare intracranial tumor,^[3]^ accounting for approximately 0.1% of intracranial tumors.^[4]^ During the embryonic period, melanocytes migrate to the intracranial pia mater along with the primitive neural crest. Because the highest density of melanocytes is usually located on the ventrolateral surface of the medulla oblongata and around the upper part of the spinal cord, which becomes the familiar site of PIMM.^[5]^ The most common symptoms are dizziness, headache, and intracranial hypertension.^[6]^ When the tumor invades the meninges and brain parenchyma, it will show meningeal irritation signs and epilepsy. When the tumor invades the skull base, it can show nerve damage symptoms, such as loss of hearing and vision, numb face, and limbs. Imaging examinations can accurately identify the location of lesions. CT scans are nonspecific, and most show a round-like high-density tumor, which can be easily confused with cerebral hemorrhage and meningioma. MRI is closely related to the content of melanin granules, and different melanin contents lead to complex MRI findings. Typical MRI characteristics include high signal intensity on T1WI and low signal intensity on T2WI.^[7]^ Final diagnosis requires histopathological examination.

The PIMM is primarily black, dark brown, blood clot-like, and has local distension growth. The adjacent meninges and skull were usually black stained. Some tumors that grew along the meninges diffused and infiltrated; therefore, the PIMM had a poor prognosis. The microscopic performance was similar to that of a malignant melanoma of the skin. Microscopic examination revealed that the tumor area was composed of spindle and oval epithelioid cells arranged in sheets and bundles. Chromatin edges of the nuclei and nucleoli were apparent. Most tumors exhibit melanin, bleeding, and necrosis. HMB-45 and Melan-A are specific indicators of PIMM, which are often diffusely positive in tumors.^[8]^ Although the expression rate is high, S-100 is often expressed in neurogenic tumors, and its specificity is low. Therefore, S-100 can be used as an auxiliary diagnostic indicator for PIMM. The Ki-67 index often exceeds 8%, and pCK, EMA, and GFAP are negative in PIMM.^[9]^ In recent years, it has been found that the mutation rate of the GNAQ gene in PIMM has been high.^[10]^ Different from malignant melanoma in skin and mucosa, PIMM has a single mutation mode and fewer mutations in BRAF, NRAS, and KIT genes. Our genetic test results are consistent with those previously reported. These studies show that GNAQ is expected to become an essential gene in the diagnosis and treatment of PIMM.

Pathological diagnosis of PIMM should be combined with clinical and imaging examinations. Therefore, we should focus on differentiating between metastatic malignant melanoma, melanocytoma, melanotic meningioma, and melanotic schwannoma. During metastatic malignant melanoma, the patient had melanoma elsewhere, such as in the skin or eyes. Most imaging findings are multiple lesions on the surface of the cerebral hemisphere or at the junction of the gray and white matter layers. Microscopically, tumor atypia is prominent, necrosis is common, and the BRAF, NRAS, and KIT gene variants are common.^[11]^ Melanocytoma is relatively benign, with uniform cell morphology, small atypia, no hemorrhage and necrosis, mitosis 0 to 1/10HPF, and no infiltration in adjacent tissues.^[12]^ Microscopically, the tumor cells of melanotic meningioma are arranged in a swirly shape, and the meningeal skin cells can be seen. The morphology of the cells was mild, and sand bodies were easily visible. IHC is often positive for EMA but negative for HMB-45 and Melan-A.^[13]^ The shape of melanotic schwannoma cells is mild and arranged in a palisade shape, which can be seen in areas A and B, but negative for HMB-45 and Melan-A.^[14]^ In addition, it needs to be distinguished from ependymoma, ganglioglioma, medulloblastoma, and other tumors, which are relatively easy to distinguish according to histological characteristics and IHC phenotype. Some PIMM contain fewer melanin particles and are easily misdiagnosed. The diagnosis of PIMM can be included in the differential diagnosis of microscopic lesions with diverse cell morphologies and obvious nucleoli.

Because of the severe side effects of chemoradiotherapy and immunotherapy, and because the drugs do not easily pass through the blood-brain barrier, the effect of these treatments is not obvious.^[15]^ The treatment of PIMM is mainly surgical resection, and postoperative chemoradiotherapy and immunotherapy can be supplemented. In terms of targeted therapy, GNAQ and its upstream and downstream pathways are expected to be therapeutic targets.

## 4. Conclusions

PIMM is a rare malignant tumor that is difficult to diagnose preoperatively. The final diagnosis require histopathological examination. Clinical manifestations and imaging characteristics can provide important information for pathological diagnosis of PIMM. GNAQ is a common variant of PIMM, and which is expected to be a therapeutic target.

## Author contributions

**Conceptualization:** Yan-Neng Xu, Wei-Li Long, Fu-Yong Chen, Xiao-Lin Huang, Jun-Xu Lu.
